# Native-valve *Staphylococcus epidermidis* endocarditis complicated by destructive pseudoaneurysm of the mitral-aortic intervalvular fibrosa: a case report and review of the literature

**DOI:** 10.1093/ehjcr/ytag434

**Published:** 2026-06-10

**Authors:** Roben Ohev Shalom, Saro Avedikian, Vishva Dev

**Affiliations:** Department of Cardiology, HCA Los Robles Medical Center, 215 W Janss Road, Thousand Oaks, CA 91360, USA; Department of Cardiology, HCA Los Robles Medical Center, 215 W Janss Road, Thousand Oaks, CA 91360, USA; Department of Cardiology, HCA Los Robles Medical Center, 215 W Janss Road, Thousand Oaks, CA 91360, USA

**Keywords:** infective endocarditis, Staphylococcus epidermidis, Coagulase-negative staphylococci, Mitral-aortic intervalvular fibrosa, Pseudoaneurysm, Transoesophageal echocardiography, Case report

## Abstract

**Background:**

Pseudoaneurysm of the mitral–aortic intervalvular fibrosa (MAIVF) represents a rare but severe complication of infective endocarditis, classically associated with highly virulent organisms such as *Staphylococcus aureus*. *Staphylococcus epidermidis*, a coagulase-negative staphylococcus traditionally considered a low-virulence commensal, is increasingly recognized as a cause of clinically significant native-valve endocarditis.

**Case summary:**

We report a 72-year-old man with native bicuspid aortic valve who presented with syncope and was found to have *S. epidermidis* bacteraemia. Transthoracic echocardiography demonstrated an aortic valve mass, but transoesophageal echocardiography (TEE) revealed extensive periannular destruction including MAIVF pseudoaneurysm, posterior aortic annular abscess, and a large mitral valve vegetation with associated thrombus. Neuroimaging confirmed multiple embolic cerebral infarcts. Despite aggressive medical therapy, the patient's clinical trajectory precluded surgical intervention, and he ultimately transitioned to comfort-focused care.

**Conclusion:**

To our knowledge, this represents one of the first reported cases of native-valve *S. epidermidis* endocarditis complicated by destructive MAIVF involvement in the absence of prosthetic material. This case underscores the underappreciated pathogenic potential of coagulase-negative staphylococci and highlights the critical role of TEE in detecting periannular complications that may be missed on transthoracic imaging.

Learning points

- To recognize that coagulase-negative staphylococci, including *Staphylococcus epidermidis*, can cause aggressive native-valve endocarditis comparable to those seen with *Staphylococcus aureus*.
- To appreciate the superior sensitivity of transoesophageal echocardiography for detecting mitral–aortic intervalvular fibrosa pseudoaneurysms and other periannular complications.
- To understand the importance of early, comprehensive imaging in patients with staphylococcal bacteraemia, particularly when clinical features suggest complicated endocarditis.

## Introduction

The mitral–aortic intervalvular fibrosa (MAIVF) is a thin, avascular fibrous structure forming the anatomic continuity between the noncoronary and left coronary cusps of the aortic valve and the anterior mitral leaflet.^1^ Owing to its limited vascular supply, the MAIVF is particularly susceptible to extension of infection from adjacent left-sided valves during infective endocarditis (IE).^2^ Periannular involvement of the MAIVF is uncommon but represents one of the most severe complications of IE, manifesting as abscess formation, pseudoaneurysm, or fistulization, with attendant risks of rupture, haemodynamic compromise, and systemic embolization.^2,3^

Destructive MAIVF involvement is classically associated with highly virulent organisms, most notably *Staphylococcus aureus* and streptococcal species, and is frequently described in patients with prior aortic valve surgery or prosthetic valve material.^3^ In contrast, CoNS—particularly *Staphylococcus epidermidis*—are traditionally regarded as low-virulence skin commensals and are often dismissed as blood culture contaminants. However, accumulating evidence from large multicentre registries indicates that CoNS can cause clinically significant native-valve IE (NVE) with extensive valvular destruction and periannular complications.^4^

We report a rare case of native-valve *S. epidermidis* endocarditis complicated by destructive MAIVF pseudoaneurysm in the absence of prosthetic material, underscoring the underappreciated virulence of this organism and the critical importance of early transoesophageal echocardiography (TEE) in patients with CoNS bacteraemia.

## Summary figure

**Figure:**
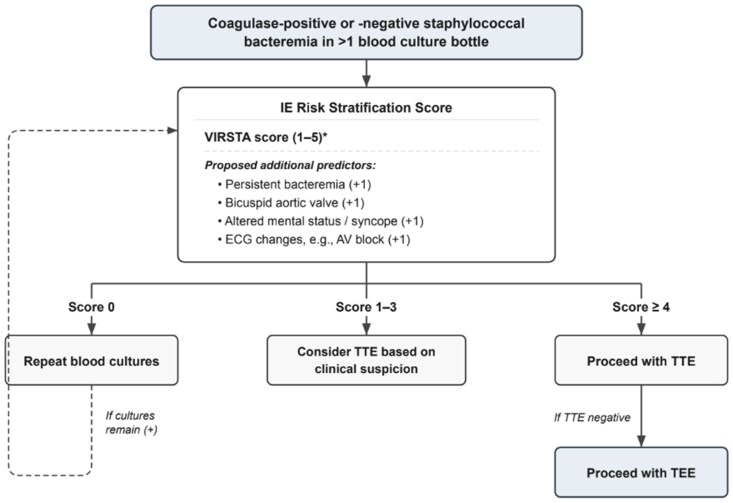


## Case presentation

A 72-year-old man with a history of hypertension, hyperlipidaemia, and recent right knee cellulitis presented to an outside hospital with recurrent syncopal episodes. Blood cultures obtained on admission grew *S. epidermidis*. Transthoracic echocardiography (TTE) demonstrated a large mobile mass on the aortic valve without apparent mitral valve pathology. Magnetic resonance imaging (MRI) of the brain revealed multiple small acute infarcts in the left middle cerebral artery and right cerebellar territories, consistent with septic embolization. The patient was transferred to our institution for further evaluation.

On arrival, physical examination revealed a grade II/VI systolic ejection murmur, diminished breath sounds at the lung bases, and healed abrasions on both knees without evidence of peripheral stigmata of IE. Laboratory studies were significant for an elevated erythrocyte sedimentation rate (74 mm/hr) and C-reactive protein (91.8 mg/L). Intravenous nafcillin was initiated for treatment of methicillin-susceptible *S. epidermidis* endocarditis.

TEE performed at our institution revealed findings substantially more extensive than those suggested by the initial TTE (*Figure 1*). A heavily calcified bicuspid aortic valve was identified with severe aortic stenosis and moderate-to-severe aortic regurgitation. A 2.5-cm broad-based homogeneous mass was visualized adjacent to the posterior aspect of the aortic valve annulus, with extension into the left atrium. This lesion demonstrated systolic expansion and diastolic collapse with colour Doppler evidence of flow communication with the left ventricular outflow tract, consistent with pseudoaneurysm formation in the MAIVF region. Additionally, a posterior periannular abscess involving approximately half of the aortic annulus circumference was identified. The mitral valve, which had appeared normal on TTE, demonstrated a probable vegetation associated with a 1.4 × 1.0 cm thrombus in a supravalvular position above the mitral annulus, with associated mild mitral regurgitation. A moderate pericardial effusion was present with fibrinous material and loculations, suggestive of pericardial involvement. Flow reversal in the descending thoracic aorta was noted, consistent with significant aortic regurgitation; however, the pattern suggested a component of chronicity, raising concern for a prolonged subclinical infectious process.

**Figure 1:**
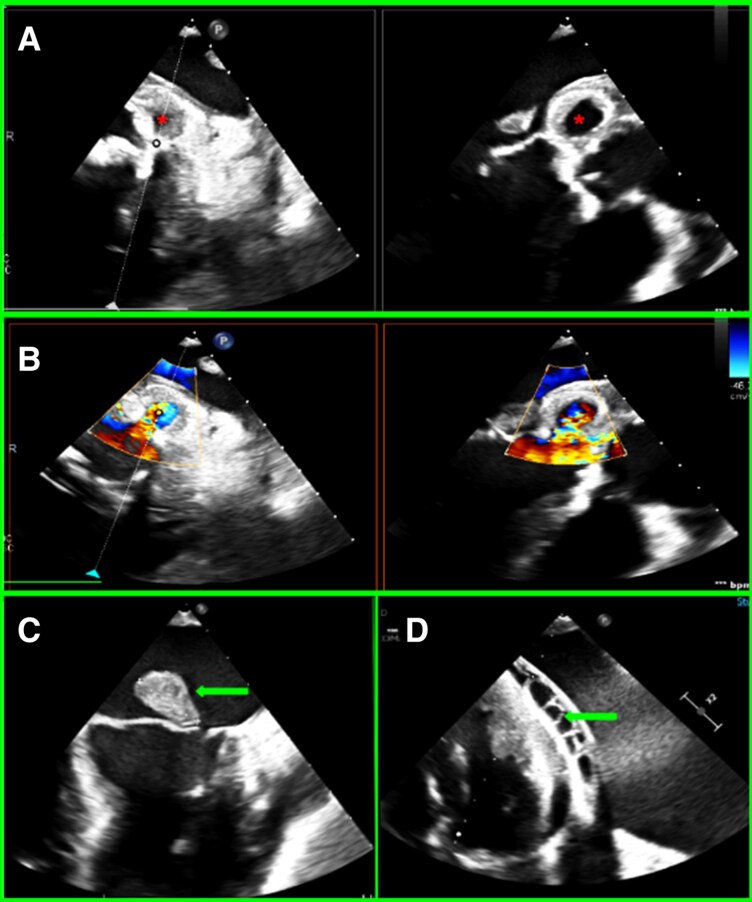
Transoesophageal echocardiographic findings. (*A*) Midoesophageal aortic valve long-axis view demonstrating a 2.5-cm broad-based mass adjacent to the posterior aortic annulus (asterisk) with extension into the left atrium, consistent with mitral–aortic intervalvular fibrosa pseudoaneurysm. Note the heavily calcified bicuspid aortic valve. (*B*) Colour Doppler imaging demonstrating flow into the pseudoaneurysm cavity during systole, confirming communication with the left ventricular outflow tract. (*C*) Mitral valve view demonstrating vegetation with associated thrombus (arrow) in supravalvular position. (*D*) Pericardial effusion with fibrinous material and loculations (arrowhead).

Repeat MRI of the brain demonstrated additional foci of restricted diffusion in the left medial temporal lobe and left anterior frontal lobe consistent with interval embolic infarcts (*Figure 2*). Computed tomography of the chest revealed moderate-to-large bilateral pleural effusions. Blood cultures obtained at our facility remained negative, likely reflecting the prior initiation of appropriate antimicrobial therapy.

**Figure 2:**
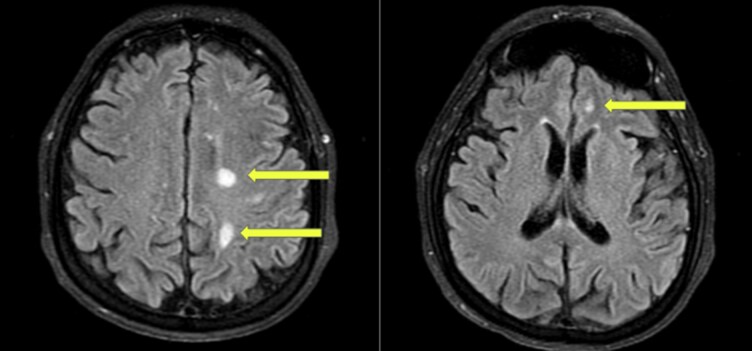
Magnetic resonance imaging of the brain. Diffusion-weighted imaging demonstrating foci of restricted diffusion in the left medial temporal lobe and left anterior frontal lobe (arrows), consistent with acute embolic infarcts secondary to septic embolization.

Despite aggressive diuretic therapy for the pleural effusions, the patient developed progressive respiratory failure requiring endotracheal intubation and mechanical ventilation. Cardiothoracic surgery evaluated the patient and determined that operative intervention carried prohibitive risk given the patient's haemodynamic instability, extensive periannular destruction, recent embolic strokes, and overall clinical trajectory. After multidisciplinary discussion with the patient's family, the decision was made to transition to comfort-focused care. The patient died shortly thereafter.

## Discussion

Although *S. epidermidis* is traditionally regarded as a low-virulence skin commensal and frequent blood culture contaminant, increasing evidence supports its role as a true pathogen in NVE. Early series characterized *S. epidermidis* NVE as uncommon and often indolent, potentially delaying diagnosis, yet capable of significant valvular destruction requiring surgery.^5^ Data from the GAMES registry, encompassing 842 coagulase-negative staphylococcal IE cases across 44 centres, demonstrate that *S. epidermidis* accounts for 77% of CoNS IE and is associated with substantial morbidity, including periannular abscess in 29% and surgical intervention in 60%.^4^

Virulence is mediated by biofilm formation—facilitated by polysaccharide intercellular adhesin and accumulation-associated protein—which promotes adherence to damaged endocardium and resistance to host defences and antimicrobials.^6^ Phenol-soluble modulins and other secreted factors further contribute to tissue injury and inflammation.^6^ Although methicillin-resistant strains possess additional virulence determinants, these mechanisms can clearly drive aggressive native-valve disease even in methicillin-susceptible isolates. The patient’s bicuspid, heavily calcified aortic valve likely created a substrate for bacterial adherence and invasive infection.^7^

Pseudoaneurysm of the MAIVF is a rare but devastating complication of IE due to the region’s avascularity and susceptibility to contiguous spread.^2^ Prior reviews identified *S. aureus* and streptococci as predominant pathogens, with no reported CoNS-associated cases.^3^ To our knowledge, only one prior report describes *S. epidermidis* IE complicated by MAIVF pseudoaneurysm, occurring postoperatively in the presence of prosthetic material.^8^ Our patient's case appears to represent one of the first reported instances of native-valve *S. epidermidis* endocarditis complicated by MAIVF pseudoaneurysm in the absence of any prosthetic material or prior cardiac surgery, expanding the recognized pathogenic potential of this organism.

This case underscores the superior sensitivity of TEE compared with TTE for detecting periannular complications of IE. Although initial TTE suggested isolated aortic valve involvement, TEE identified MAIVF pseudoaneurysm, posterior annular abscess, and a large mitral vegetation with thrombus—findings not visualized on transthoracic imaging. This aligns with prior data showing TTE sensitivity of 28%–50% for periannular abscess vs. 78%–90% for TEE.^9^ The posterior location and complex anatomy of the MAIVF further limit TTE visualization. Accordingly, current guidelines recommend TEE when IE is suspected or clinical concern persists.^10^

The VIRSTA score, developed to identify patients with *S. aureus* bacteraemia at high risk for IE and guide TEE use, has demonstrated excellent sensitivity (98.9%–100%) and negative predictive value (98.8%–99.5%) for ruling out endocarditis.^11^ While this tool was designed and validated specifically for *S. aureus* bacteraemia, our case illustrates that similar risk stratification approaches may be worthy of future investigation in CoNS bacteraemia, although such applications remain hypothetical and unvalidated.

Our patient had multiple high-risk features that would have prompted early TEE, highlighting the potential utility of a modified VIRSTA calculator for CoNS bacteraemia (Summary figure). Other factors to consider that have been well documented in the literature to predispose to endocarditis include persistent bacteraemia,^12^ bicuspid aortic valve,^13^ presentation with altered mental status/syncope,^14^ or ekg changes.^15^

Given the demonstrated capacity of CoNS to cause aggressive native-valve disease with periannular complications, we propose that clinicians maintain a high index of suspicion for complicated IE in patients with CoNS bacteraemia, particularly those with pre-existing valvular abnormalities, persistent bacteraemia, or embolic events. Early TEE should be strongly considered in such patients, as TTE may significantly underestimate disease extent.

## Supplementary Material

ytag434_Supplementary_Data

## Lead author biography

Dr. Roben Ohev Shalom is a second-year internal medicine resident and aspiring cardiologist at Los Robles Medical Center in Thousand Oaks, CA. He earned his Doctor of Medicine degree from Oakland University William Beaumont School of Medicine in Auburn Hills, MI. Prior to that, he graduated Summa Cum Laude from UCLA with bachelors of science in biochemistry. His academic interests include cardiovascular imaging, echocardiography, interventional cardiology and medical education. He is particularly interested in the use of advanced cardiac imaging to guide complex coronary and structural heart interventions.

## Statements

The data underlying this article are available in the article and in its online Supplementary material. Additional data underlying this article will be shared on reasonable request to the corresponding author.

Written informed consent was obtained from the patient for publication of this case report and any accompanying images. All efforts were made to protect the patient’s identity, in accordance with COPE guidelines.
